# Protecting effects of smoking against COVID-19: a community-based retrospective cohort study in middle- and older-aged adults

**DOI:** 10.1007/s11739-024-03713-5

**Published:** 2024-08-20

**Authors:** Xiaomeng Hou, Fulin Zheng, Likun Lu, Zhenjie Wang, Xuefeng Ni

**Affiliations:** 1 Department of Health Care, Peking Union Medical College Hospital, Chinese Academy of Medical Sciences and Peking Union Medical College, Beijing, China; 2 Department of Radiology, Peking Union Medical College Hospital, Chinese Academy of Medical Sciences and Peking Union Medical College, Beijing, China

**Keywords:** Coronavirus disease 2019 (COVID-19), Smoking, Risk factor, Nicotine replacement therapy, Angiotensin-converting enzyme 2 (ACE2)

## Abstract

On December 7, 2022, China switched from dynamic zeroing strategy against severe acute respiratory syndrome coronavirus 2 (SARS-CoV-2) to reopening. A nationwide SARS-CoV-2 epidemic emerged rapidly. The effect of smoking on SARS-CoV-2 infection remains unclear. We aimed to retrospectively investigate the relationship between smoking and coronavirus disease 2019 (COVID-19) using a community-based cohort of smokers and non-smokers. We included participants from a pre-pandemic cohort with a prolonged follow-up period. Data on smoking status, body mass index, and history of other diseases were collected from health examination and consultation clinic records. Cox regression analysis was used to identify the relationship between groups and SARS-CoV-2 infection over time. We analysed 218 male patients with varied smoking statuses (46.3% current or ex-smokers; average age 68.63 ± 9.81 years). Two peaks in the epidemic were observed following the December 2022 outbreak. At the end of the second peak, non-smokers, current smokers, and ex-smokers had primary infection rates increase to 88.0%, 65.1%, and 81.0%, respectively, with a significant difference between the groups. Current smoking significantly protected against SARS-CoV-2 infection (HR 0.625, 95% CI 0.402–0.970, *p* = 0.036). Further analyses showed that the prevalence of pneumonia in the unvaccinated, older, diabetic, and non-smoking groups was significantly higher than that in the other groups (*p* < 0.05). Our study suggests a potential association between smoking and a reduced risk of SARS-CoV-2 infection and pneumonia. This indicates that nicotine and ACE2 play important roles in preventing COVID-19 and its progression. We suggest smokers use nicotine replacement therapy during hospitalization for COVID-19.

## Introduction

Until December 31, 2023, there have been 773,449,299 confirmed cases of coronavirus disease 2019 (COVID-19), with 6,991,842 deaths reported globally to the WHO [1]. The COVID-19 pandemic began in late 2019 and considerably impacted China. Since then, China has effectively controlled the pandemic, achieving a state of “dynamic zero” cases over 3 years. Factors such as widespread use of vaccines, expansion of medical resources, and weakened virulence of the virus have contributed to reducing deaths and harm caused by COVID-19 [2–4]. On December 7, 2022, China changed its management of COVID-19, switching from a dynamic zeroing stage approach to reopening. A nationwide epidemic of severe acute respiratory syndrome coronavirus 2 (SARS-CoV-2) emerged within a month. According to a report from the Chinese Center for Disease Control and Prevention, the number of hospitalized patients with COVID-19 in China reached a peak of 1.625 million on January 5, 2023, and has since declined. As of February 13, 2023, the number has decreased to 26,000, a 98.4% reduction from the peak [5]. This kind of outbreak on the basis of zero to go hand in hand, resulting in the number of infections peaked in a very short time, provides an excellent opportunity to observe the risk and protective factors associated with infection.

Compared to the well-established adverse effects of smoking on cancer, cardiovascular and cerebrovascular diseases, as well as chronic obstructive pulmonary disease, the risk of developing COVID-19 in patients with different smoking status remains paradoxical. Studies have reported mixed findings regarding the relationship between smoking and COVID-19 during the pandemic. A spatial analysis of 175 countries showed that the percentage of smokers was significantly and inversely associated with COVID-19 in Asian, Arab, and Pacific countries [6]. Similarly, an ecological study found a statistically significant negative association (*p* = 0.001) between smoking prevalence and the prevalence of COVID-19 in 38 European nations [7]. According to primary care records in the UK, it was found that current smoking was associated with an 11% lower chance of COVID-19 related death among 17.3 million adults [8]. However, several studies aimed to demonstrate that smoking increases the risk of COVID-19 and its progression [9–17]. In a meta-analysis of 22,939 patients with COVID-19, 2914 (12.7%) were current and former smokers. Among them, 33.5% of smokers experienced disease progression compared to 21.9% of non-smokers [17]. The evidence on the effects of smoking on COVID-19 infection and severe complications comes from three main sources: macroepidemiology, patient cohorts infected with COVID-19, and systematic reviews. During the pandemic, the latter two categories collected and assessed data from patients with COVID-19 and did not accurately represent uninfected populations. To date, no study has accurately represented the prevalence of SARS-CoV-2 infections, as well as non-infections, among smokers in general population. Therefore, this study used a community-based follow-up cohort of smokers and non-smokers established in a health care clinic prior to the pandemic to retrospectively observe the distribution characteristics of primary infection of SARS-CoV-2 among middle-aged and older community residents in China after three years of silence. Our aim was to investigate the potential association between smoking and a decreased risk of SARS-CoV-2 infection and pneumonia, as well as to explore possible preventive and therapeutic mechanisms.

## Materials and methods

### Ethical considerations

This study was approved by the Ethics Committee. The institutional review board waived the requirement for informed consent because the data were anonymous and this study was retrospective. This study was conducted in accordance with the principles of the Declaration of Helsinki.

### Study setting

This retrospective cohort study investigated the association between smoking status and COVID-19. Participants were divided into three groups: non-smokers, current smokers, and ex-smokers. The observation period was from December 7, 2022, to June 30, 2023.

### Study population

**Fig. 1:**
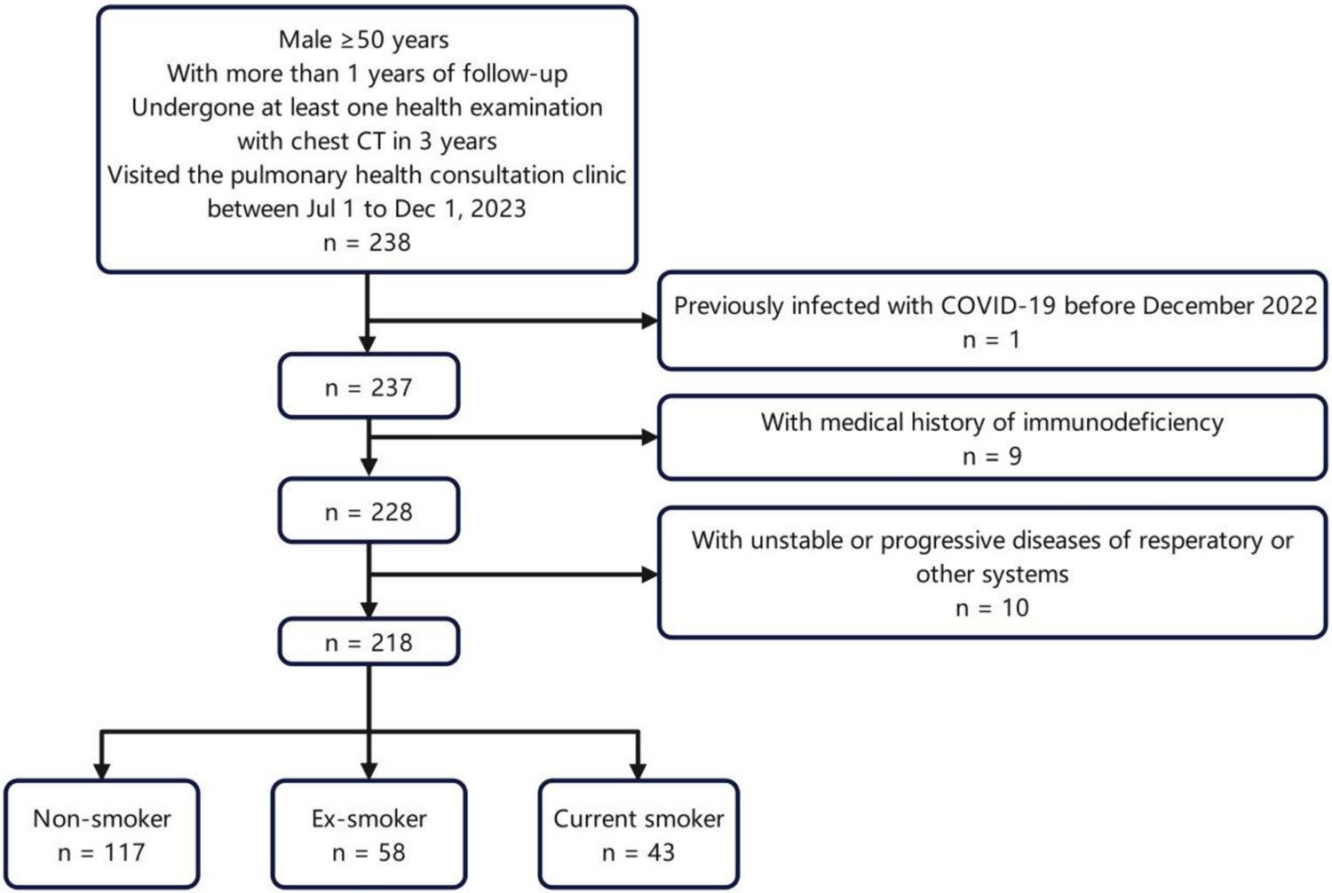
The enrolment and exclusion of the study population

Eligible smokers and non-smokers who visited the pulmonary health consultation clinic between July 1, 2023, and December 1, 2023 were consecutively enrolled. Each patient had assessed for the presence of initial COVID-19 infection, smoking status, and COVID-19 vaccination history. The inclusion criteria were as follows: (1) male subjects aged > 50 years, (2) regularly followed up at the health care centre for more than 1 year, (3) and undergone at least one comprehensive health examination with formal history collection and chest computed tomography (CT) at the health care centre within the past 3 years. The exclusion criteria were as follows: (1) prior SARS-CoV-2 infection before December 2022, (2) history of immunodeficiency, including diagnosed haematological and autoimmune diseases, radiotherapy and chemotherapy for malignant tumours, and immunosuppressive therapy, (3) unstable conditions during the peri-observation period, such as progressive tumours, acute myocardial infarction, chronic active infections, and acute exacerbation of chronic diseases, and (4) patients with unstable or progressive respiratory system disease during the peri-observation period, including asthma with unstable symptoms, high-risk of exacerbation of chronic obstructive pulmonary disease (ECOPD), acute pulmonary embolism, interstitial lung disease (ILD) requiring antifibrotic or immunosuppressive therapy, diffuse bronchiectasis, or other lesions. A flowchart of the enrolment and exclusion process of the study population is shown in Fig. 1.

### Data collection and assessment

Data on smoking status, body mass index (BMI), and history of other diseases such as diabetes and hypertension were collected from health screening information reports. COVID-19 history was collected from the records of the pulmonary health consultation clinic.

Non-smokers were defined as those who had not smoked in their lifetime and were not smoking at the time of consultation. Current smokers were defined as those who smoked regularly at the time of consultation. Ex-smokers were those who had smoked regularly in their lifetime, but not at the beginning of the observation period (December 7, 2022) for at least 6 months.

The basic pulmonary conditions of the enrolled patients were divided into mildly abnormal and normal groups according to CT findings. Mild abnormalities included local tubular bronchiectasis, pulmonary nodules after wedge resection, obsolete lesions after curing tuberculosis or other pulmonary injuries, and mild stable interstitial changes that did not reach a diagnosis of ILD.

The identification of SARS-CoV-2 infection required simultaneous respiratory and systemic symptoms with evidence of antigen or nucleic acid positivity. COVID-19 pneumonia was defined as new lung shadows appearing within 3 weeks of SARS-CoV-2 infection that was not attributable to other pulmonary infections. Therefore, occult infections or asymptomatic pneumonia may have existed among uninfected patients or non-pneumonia COVID-19 identified in this study. Primary infection refers to an initial encounter with SARS-CoV-2. Our study exclusively recorded and analysed primary infections among the participants.

## Statistical analysis

The relationship between smoking status and COVID-19 infection was analysed using SPSS Modeller (V18.4, IBM, Arming, NY, USA). Normally distributed quantitative variables are shown as means ± SD, and the categorical data are presented as frequencies and percentages. Analysis of variance was used to compare measured data, and the χ2 test was used for categorical count data. Differences were considered statistically significant at *p* < 0.05. Cox regression analysis was used to identify the relationship between the variables and SARS-CoV-2 infection with prolonged exposure times. Survival time in the Cox regression analysis was defined as the duration of remaining uninfected, from the onset of the pandemic on December 7, 2022, to the participant's initial COVID-19 diagnosis.

## Results

### Basic characteristics based on smoking status

A total of 218 patients with different smoking status were included. There were 117 (53.7%) participants in the non-smoking group, 43 (19.7%) in the current smoking group, and 58 (26.6%) in the ex-smoking group. The average age was 68.63 ± 9.81 years, ranging from 51 to 94 years. Twelve (5.5%) patients had no SARS-CoV-2 vaccination, 201 (92.2%) had one to three doses, and five (2.3%) had four doses. Mean BMI was 26.00 for smokers, 25.85 for non-smokers, and 26.19 for ex-smokers. Seventy-four (33.9%) participants had mildly abnormal lung conditions, whereas 144 (66.1%) had normal lung conditions. In total, 110 (50.5%) and 64 (29.4%) patients had hypertension and diabetes, respectively. The distribution of the basic characteristics among the different smoking groups is shown in Table 1.

**Table 1:** The distribution of basic characteristics among groups with different smoking status

	Non-smokers *n* = 117	Current smokers *n* = 43	Ex-smokers *n* = 58	Total *n* = 218	F or χ2	*p* value
Age	69.92 ± 10.91	64.49 ± 7.73	69.09 ± 7.99	68.63 ± 9.81	5.092	0.007
50–59	21 (17.9%)	12 (27.9%)	6 (10.3%)	39 (17.9%)		
60–74	59 (50.4%)	26 (60.5%)	36 (62.1%)	121 (55.5%)		
≥ 75	37 (31.6%)	5 (11.6%)	16 (27.6%)	58 (26.6%)		
BMI	25.85 ± 2.82	26.00 ± 2.24	26.19 ± 2.61	25.97 ± 2.66	0.319	0.728
< 24	31(26.5%)	6 (14.0%)	8 (13.8%)	45 (20.6%)		
≥ 24, < 28	62 (53.0%)	27 (62.8%)	38 (65.5%)	127 (58.3%)		
≥ 28	24 (20.5%)	10 (23.3%)	12 (20.7%)	46 (21.1%)		
Vaccination					2.417	0.660
0 dose	5 (4.3%)	3 (7.0%)	4 (6.9%)	12 (5.5%)		
1–3 doses	108 (92.3%)	40 (93.0%)	53 (91.4%)	201 (92.2%)		
4 doses	4 (3.4%)	0 (0.0%)	1 (1.7%)	5 (2.3%)		
Lung condition					6.572	0.037
Normal	70 (59.8%)	35 (81.4%)	39 (67.2%)	144 (66.1%)		
Mild abnormal	47 (40.2%)	8 (18.6%)	19 (32.8%)	74 (33.9%)		
Diabetes					1.818	0.403
No	86 (73.5%)	31 (72.1%)	37 (63.8%)	154 (70.6%)		
Yes	31 (26.5%)	12 (27.9%)	21 (36.2%)	64 (29.4%)		
Hypertension					9.120	0.010
No	54 (46.2%)	30 (69.8%)	24 (41.4%)	108 (49.5%)		
Yes	63 (53.8%)	13 (30.2%)	34 (58.6%)	110 (50.5%)		

### Prevalence of primary COVID-19 in China from December 2022 to June 2023

**Fig. 2:**
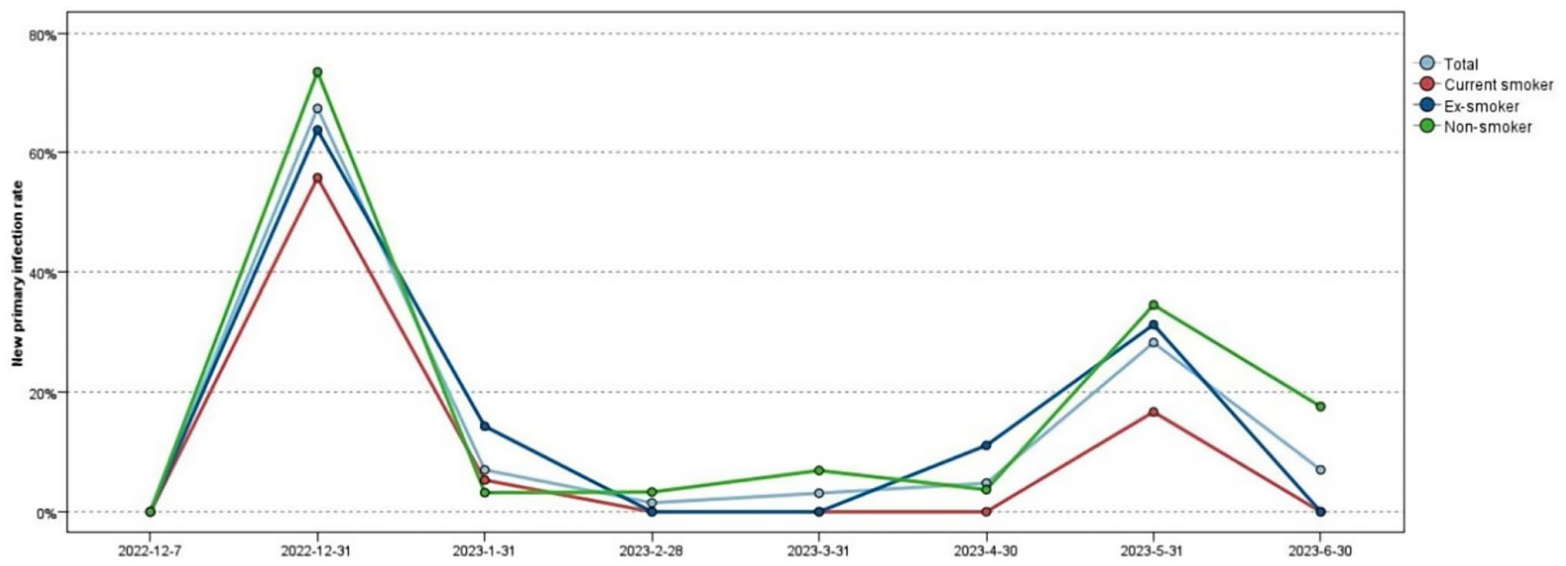
The new primary infection rates over time among different groups. The new primary infection rate was calculated as the ratio of all new primary infections in the current month to the number of remaining uninfected individuals at the beginning of the month

As per official forecasts, China experienced two peaks in COVID-19 since the outbreak in December 2022. The first peak occurred from December 2022 to January 2023, and the second peak emerged around May 2023, encompassing both primary and secondary COVID-19 infections. This study showed that at the end of the first peak, the primary infection rates of the current smokers, ex-smokers, non-smokers, and entire population were 58.1%, 69.0%, 74.4%, and 69.7%, respectively. At the end of the second peak, the primary infection rates in the above groups reached 65.1%, 81.0%, 88.0%, and 81.7%, respectively, and the difference among the groups was significant (*p* = 0.004). The changes in the new primary infection rates over time among the different groups are shown in Fig. 2.

### Cox survival analysis of factors associated with overall uninfected

**Fig. 3:**
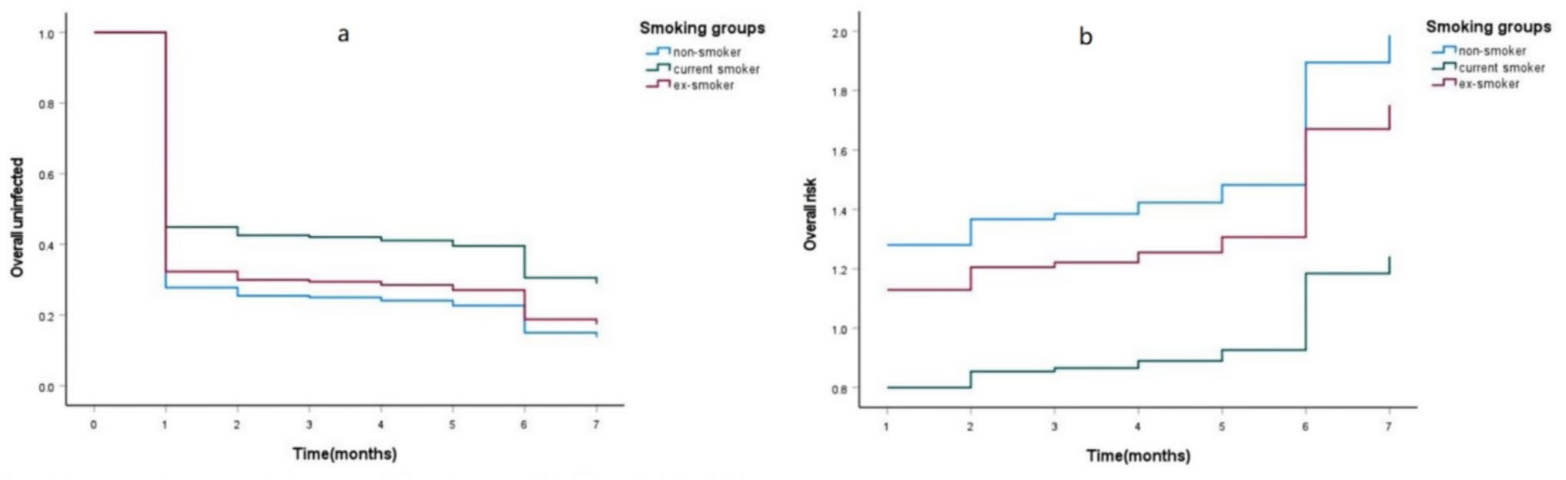
The overall uninfected (**a**) and the overall risk of infection (**b**) over time among different smoking groups in Cox regression

Univariate and multivariate Cox regression analyses were performed to determine the factors associated with SARS-CoV-2 infection over time. Only current smoking status was confirmed as an independent negative prognostic factor for SARS-CoV-2 infection (*p* < 0.05; Table 2). The proportion of uninfected individuals and the risk of infection over time between the different smoking groups are shown in Fig. 3.

**Table 2:** Univariate and multivariate Cox regression of factors associated with overall uninfected

Variables	Univariable cox regression			Multivariable cox regression		
	HR	95.0% CI	*p* value	HR	95.0% CI	*p* value
Smoking group						
Non-smoking (control)	–	–	–	–	–	–
Current smoking	.655	.430–.996	.048	.625	.402–.970	.036
Ex-smoking	.884	.626–1.248	.483	.882	.619–1.255	.485
Lung condition						
Normal (control)	–	–	–	–	–	–
Mild abnormal	.944	.690–1.291	.717	.901	.648–1.253	.537
Vaccination						
0 dose (control)	–	–	–	–	–	–
1–3 doses	1.210	.618–2.367	.578	1.163	.567–2.387	.681
4 doses	.808	.219–2.984	.749	.603	.150–2.423	.476
Body mass index						
< 24 (control)	–	–	–	–	–	–
≥ 24, < 28	1.039	.709–1.524	.843	1.096	.743–1.616	.645
≥ 28	1.083	.684–1.714	.734	1.193	.742–1.916	.467
Hypertension						
None (control)	–	–	–	–	–	–
Yes	1.157	.861–1.553	.333	1.126	.814–1.558	.473
Diabetes						
None (control)	–	–	–	–	–	–
Yes	1.003	.726–1.385	.987	.976	.689–1.383	.893
Age						
50–59 (control)	–	–	–	–	–	–
60–74	.811	.548–1.202	.297	.739	.491–1.113	.148
≥ 75	.904	.584–1.402	.653	.799	.488–1.307	.371

### Distribution of COVID-19 pneumonia in groups with different variables

We further analysed the distribution of the incidence of COVID-19 pneumonia among the different variable groups (Table 3). Among the participants, 18 (8.3%) were diagnosed with COVID-19 pneumonia. By comparing different variables, we found that the prevalence of pneumonia in current smokers (0%) was significantly lower than that in non-smokers (*p* = 0.047). Among various age groups, the prevalence of pneumonia in the ≥ 75 year old group (19%) was significantly higher than that in younger age groups (*p* = 0.002). Among different vaccination groups, the prevalence of pneumonia in the non-vaccinated group (33.3%) was significantly higher than that in the other two groups (*p* = 0.004). The prevalence of pneumonia in patients with diabetes (14.1%) was significantly higher than that in patients without diabetes (*p* = 0.045).

**Table 3:** The distribution and comparison of COVID-19 pneumonia in different variable groups

Characteristic	Pneumonia			
	None (%)	Yes (%)	χ2	*p* value
Smoking group			6.136	0.047
Non-smoking	103 (88.0)	14 (12.0)		
Current smoking	43 (100.0)	0 (0.0)		
Ex-smoking	54 (93.1)	4 (6.9)		
Age			12.002	0.002
50–59	37 (94.9)	2 (5.1)		
60–74	116 (95.9)	5 (4.1)		
≥ 75	47 (81.0)	11(19.0)		
Vaccination			10.854	0.004
0 dose	8(66.7)	4(33.3)		
1–3 doses	187(93.0)	14(7.0)		
4 dose	5(100.0)	0(0.0)		
Body mass index			1.439	0.487
< 24	40 (88.9)	5 (11.1)		
≥ 24, < 28	116 (91.3)	11 (8.7)		
≥ 28	44 (95.7)	2 (4.3)		
Lung condition			0.003	0.954
Normal	132(91.7)	12(8.3)		
Mild abnormal	68(91.9)	6(8.1)		
Hypertension			0.204	0.652
None	100(92.6)	8(7.4)		
Yes	100(90.9)	10(9.1)		
Diabetes			4.031	0.045
None	145(94.2)	9 (5.8)		
Yes	55(85.9)	9 (14.1)		

## Discussion

Smoking poses a significant threat to public health and places a substantial economic burden on society. In 2019, China had 341 million smokers, constituting 24% of its population and representing one-third of global consumption. Smoking prevalence was notably high, reaching 49.7% in males and 3.54% in females [18]. In this study, the prevalence of smoking in males (46.3%) and hypertension in older individuals (50.5%) was consistent with the average levels in China [19]. However, the effect of smoking on COVID-19 remained unknown. This study therefore focused on the relationship between smoking and susceptibility to COVID-19 in relatively healthy male individuals older than 50 years, and suggests a potential association between smoking and a reduced risk of SARS-CoV-2 infection and pneumonia.

During the initial phase of the COVID-19 outbreak, a low prevalence of current smoking (6.5%, 95% CI 4.9–8.2%) was observed among patients with COVID-19 in Wuhan, China, constituting approximately one-fourth of the population smoking prevalence [20]. A systematic review of published articles indicated a lower prevalence of smoking in patients with COVID-19 than the regional average [21]. Globally, a spatial analysis involving 175 countries [6] and an ecological study across 38 European nations [7] found a significant negative association between smoking and COVID-19. However, based on the long-standing reputation that smoking is harmful to human health, some researchers have questioned the positive effects of smoking on protection against COVID-19 and disease progression [16]. Our study suggested that current smoking significantly protects against SARS-CoV-2 infection (HR 0.625, 95%CI 0.402–0.970, *p* = 0.036). Even ex-smoking status showed a trend of effect on protection against COVID-19 (HR 0.882, 95%CI 0.619–1.255, *p* = 0.485). The participants in this study were from a follow-up cohort of health care clinics. Detailed smoking history, underlying diseases, and COVID-19 vaccination status were collected during the long-term follow-up period, effectively eliminating potential unreliable factors in the context of emergency infections.

SARS-CoV-2 activates the innate immune system, leading to the release of numerous cytokines, including IL-6. This cytokine surge can increase vascular permeability, causing fluid and blood cells to migrate into the alveoli, resulting in dyspnoea and respiratory failure [22]. Nicotine has anti-inflammatory and immunomodulatory effects. It is an agonist of the cholinergic anti-inflammatory pathway that regulates host immune and inflammatory responses [23, 24]. Nicotine inhibits the production of pro-inflammatory cytokines such as TNFα, IL-1, and IL-6, without inhibiting the production of anti-inflammatory cytokines such as IL-10 [23]. Farsalinos et al. propose a hypothesis that COVID-19 appears to become a disease of the nicotinic cholinergic system. The extensive replication of the virus will disrupt the cholinergic anti-inflammatory pathway, leading to the development of a cytokine storm, with acute lung injury resulting in ARDS, coagulation disturbances, and multiorgan failure. Nicotine could potentially restore the function of the cholinergic anti-inflammatory system and prevent the cytokine storm [25]. Subsequent in silico studies have revealed a range of complexes with cholinergic agonists that may have the potential to inhibit the binding of the SARS-CoV-2 Spike glycoprotein to nicotinic acetylcholine receptors (nAChRs), thus averting dysregulation of the Nicotinic Cholinergic System [26, 27]. Based on the results of this study and the effective anti-inflammatory mechanism of nicotine, we suggest that current smokers should use nicotine replacement therapy during hospitalization due to COVID-19 to avoid further damage caused by the storm of inflammatory factors.

In smokers, the recently observed angiotensin-converting enzyme 2 (ACE2) upregulation may be an important and beneficial defence mechanism [20, 28]. ACE2 serves as a cellular entry receptor for SARS-CoV-2 [29]. Active cigarette smoking and COPD upregulate ACE2 expression in the lower airways [28]. Increased ACE2 expression and activity has also been observed in the serum of smokers [30]. ACE2 has indirect anti-inflammatory and anti-oxidative effects and may improve outcomes of critically ill patients [20, 31]. Lutchman et al. discussed the relationship among nicotine, ACE2, and SARS-CoV-2 and concluded that soluble ACE2 may even serve as a bait to neutralize the spike protein on the surface of SARS-CoV-2, offering an effective treatment for COVID-19 [32, 33]. However, this idea contradicts the well-known theory that downregulation of ACE2 reduces susceptibility to SARS-CoV-2 infection [34]. The low prevalence of infection in current smokers in this study indicates that although ACE2 is a pathway for viral entry, upregulation of the receptor in the serum or lungs may also play an important role in defence against the virus. This could explain the protective effect in both current smokers and ex-smokers, as ex-smokers may retain upregulated ACE2 in the serum or lungs.

SARS-CoV-2 mainly invades through the respiratory tract, and the innate immunity of the lung is the first recognition and defence barrier [35]. However, few studies have examined the correlation between mild lung abnormalities, including minor interstitial or parenchymal lung structure changes, and COVID-19 prognosis. The participants included in this study with a long-term follow-up period allowed us to assess this relationship. In total, 74 (33.9%) participants in our study exhibited mild lung abnormalities: 36 (48.6%) exhibited parenchymal lung structural changes and 38 (51.4%) exhibited mild stable interstitial changes. Our analyses found that these mild lung lesions did not significantly increase the risk of SARS-CoV-2 infection and pneumonia.

SARS-CoV-2 vaccines played a crucial role in controlling the COVID-19 pandemic in China [4]. The COVID-19 vaccine is efficient in preventing the development of severe and critical COVID-19 pneumonia [36]. The rate of protection against severe illness was 90.15% in patients over 60 years old [37]. Our study findings suggest a significant protective effect of vaccines in preventing pneumonia, which aligns with the previous findings. Increasing age, obesity, and general co-morbidities are associated with intensive care unit admission, need for invasive ventilation, and mortality [38–40]. Our study further analysed the differences in the prevalence of COVID-19 pneumonia among different groups for each variable. The prevalence of pneumonia in the unvaccinated (*p* = 0.004), older (*p* = 0.002), diabetic (*p* = 0.045), and non-smoking (*p* = 0.047) groups was significantly higher than that in the other groups.

This study had several limitations. First, because the data were collected from patients who visited the clinic for regular follow-up within a certain period, selection bias may have occurred. Additionally, the study’s limitation to male participants may impact the generalizability of the findings to females. Second, data collection was limited to a single centre, ensuring quality control but greatly limiting the study’s scale. Unaccounted confounding factors like socioeconomic status and lifestyle linked to smoking may affect outcomes. However, due to the study’s retrospective nature, unrecorded information couldn’t be retrieved for analysis. Third, subjective bias in data evaluation and collection was challenging to eliminate in this retrospective study. To mitigate bias, we opted to sample from the existing follow-up cohort and avoided relying on a single doctor for data evaluation. This study has two strengths. First, it accurately depicts the primary infection rates of the two epidemic peaks in the Chinese since the end of 2022. Second, detailed community-level data were used to demonstrate the protective effect of smoking against SARS-CoV-2 infection.

## Conclusion

Our study findings suggest a potential association between smoking and a reduced risk of SARS-CoV-2 infection and pneumonia among middle-aged and older individuals in the general population during the COVID-19 pandemic in China. Two potential mechanisms were proposed to explain these observations. Firstly, the immunomodulatory effects of nicotine may have a protective impact against the virus. Additionally, the defence or neutralizing effect of ACE2 in serum and lungs may play a role in preventing viral invasion. It is important to note that while these findings are intriguing, they should be interpreted with caution due to the well-established health risks associated with smoking. We therefore suggest that smokers use nicotine replacement therapy during hospitalization for COVID-19. Furthermore, we recommend additional investigation into whether ACE2 can effectively neutralize SARS-CoV-2 at the laboratory level. Simultaneously, we anticipate prospectively including more comprehensive sociodemographic confounders and observing the long-term performance of our smoking cohort in later COVID-19 prevalence.

## Data Availability

The data presented in this study are available on request from the corresponding author.
